# The Correlation Between Collagen Types and Ultrasound Feature Score in Evaluating the Vulnerability of Carotid Artery Plaque

**DOI:** 10.3389/fcvm.2021.756424

**Published:** 2021-11-02

**Authors:** Ruijun Han, Yanhong Yan, Yafang Ding, Yabo Huang, Peng Zhou, Pinjing Hui

**Affiliations:** ^1^Department of Stroke Center, The First Affiliated Hospital of Soochow University, Suzhou, China; ^2^Department of Ultrasound, Ren Ji Hospital, Shanghai Jiao Tong University School of Medicine, Shanghai, China

**Keywords:** collagen, arteriosclerosis, plaque, ultrasound, carotid artery

## Abstract

**Objectives:** To investigate whether ultrasound score has clinical value in identifying carotid artery-vulnerable plaque and the impacts of collagen distribution on the stability of plaque.

**Materials and Methods:** Standard carotid artery ultrasound examinations were performed in 51 patients with carotid artery plaques before carotid endarterectomy. Hematoxylin-eosin staining and Sirius red–picric acid staining of plaque sections were performed to analyze the pathological features and collagen distribution. All plaques were classified into vulnerable and stable groups by pathological features. Ultrasound scores, cap thickness, and the ratios of different collagen types were recorded and analyzed between two groups and different parts of plaques.

**Results:** Ultrasound scores of the vulnerable group were higher than those of the stable group (4.35 ± 1.23 vs. 2.09 ± 1.04, *P* = 0.001). AUC was 0.894 (best cutoff point three) in differentiating vulnerable and stable plaques. Compared with the stable group, the fibrous caps of the vulnerable group were thinner (*P* = 0.012); the area ratios of collagen type I to all collagen in the vulnerable group were lower (*P* = 0.033); however, the area ratios of collagen type IV to all collagen were higher (*P* = 0.026). Compared with downstream shoulders, the ultrasound scores of upstream shoulders of plaque were higher (*P* = 0.001), the fibrous caps of upstream shoulders were thinner (*P* = 0.001), and the area ratios of collagen type I to all collagen were lower (*P* = 0.022).

**Conclusion:** Ultrasound score could have a clinical value in identifying vulnerable carotid artery plaque, and the collagen distribution could impact the stability of plaques, especially collagen type I and type IV. The results also prompted that the upstream shoulders were more vulnerable than the downstream shoulders.

## Introduction

Ischemic stroke is very harmful to human health because of its high disability rate and mortality. Global burden of disease study 2016 showed that cerebrovascular diseases were the second cause of disability worldwide, and acute ischemic stroke occupied more than two-third of cases in all cerebrovascular diseases (1). Otherwise, the incidence of stroke increased year by year, which has become one of the three leading causes of death (2–4). Previous studies indicated that carotid atherosclerotic plaque was one of the most important risk factors for acute ischemic stroke (5, 6). More than 30% of acute ischemic stroke was caused by the rupture of vulnerable carotid arterial plaque and embolization besides simple cervical vascular stenosis (7, 8). Vulnerable plaques were defined by Naghavi et al. as atherosclerotic plaques prone to thrombosis, with a tendency to rupture or rapid progression (9). Most carotid arterial plaque ruptures have no precursors, which are difficult to be predicted and diagnosed. Therefore, the identification of atherosclerotic vulnerable plaque, effective prevention of acute stroke, and reduction of its incidence have become urgent public health problems.

Carotid artery ultrasound is widely used in diagnosing vascular plaque. It has high sensitivity in detecting plaque, which can evaluate not only carotid stenosis but also plaque composition and surface integrity by echogenicity changes (10, 11). A few studies have focused on the relationship between the ultrasound results and the different components that can affect plaque stability, such as neovascular distribution and lipid core ratio. Few studies have focused on the relationship between different types of collagens and the vulnerability of plaque, especially carotid artery plaque. But none of them has studied the relationship between the ultrasound results and the different types of collagens.

Our study aimed to analyze the different types of collagen ratio in carotid artery plaque and different parts of each plaque: upstream shoulder and downstream shoulder, which could have the clinical value in vulnerable plaque diagnosis. We also assessed the carotid artery ultrasound's ability in identifying vulnerable plaque by comparing the ultrasound scores and pathological features. In addition, the relationship between the different types of collagen ratio and ultrasound score was also researched.

## Materials and Methods

### Patients

This retrospective study period was from January 2017 to July 2019, in accordance with the Code of Ethics of the World Medical Association (Declaration of Helsinki) for experiments involving human's Declaration and also approved by the local ethics committee (No. 2019124). Informed consent was obtained from all involved patients.

Inclusion criteria were as follows: 1. Patients with carotid artery plaque; 2. standard carotid artery ultrasound examination; and 3. carotid endarterectomy (CEA) operation within 24 h after the ultrasound examination. The indication of CEA operation followed North American Symptomatic Carotid Endarterectomy (NASCET) criteria: asymptomatic patients with carotid artery luminal stenosis ≥70% or symptomatic patients with luminal stenosis ≥50% (12).

Exclusion criteria were as follows: 1. incomplete carotid artery plaque specimen; 2. failure to stain the plaque (hematoxylin-eosin or Sirius red staining); 3. lack of standard carotid artery ultrasound images; and 4. lack of complete medical history or serum test information.

### Conventional Carotid Artery Ultrasound

**Figure 1:**
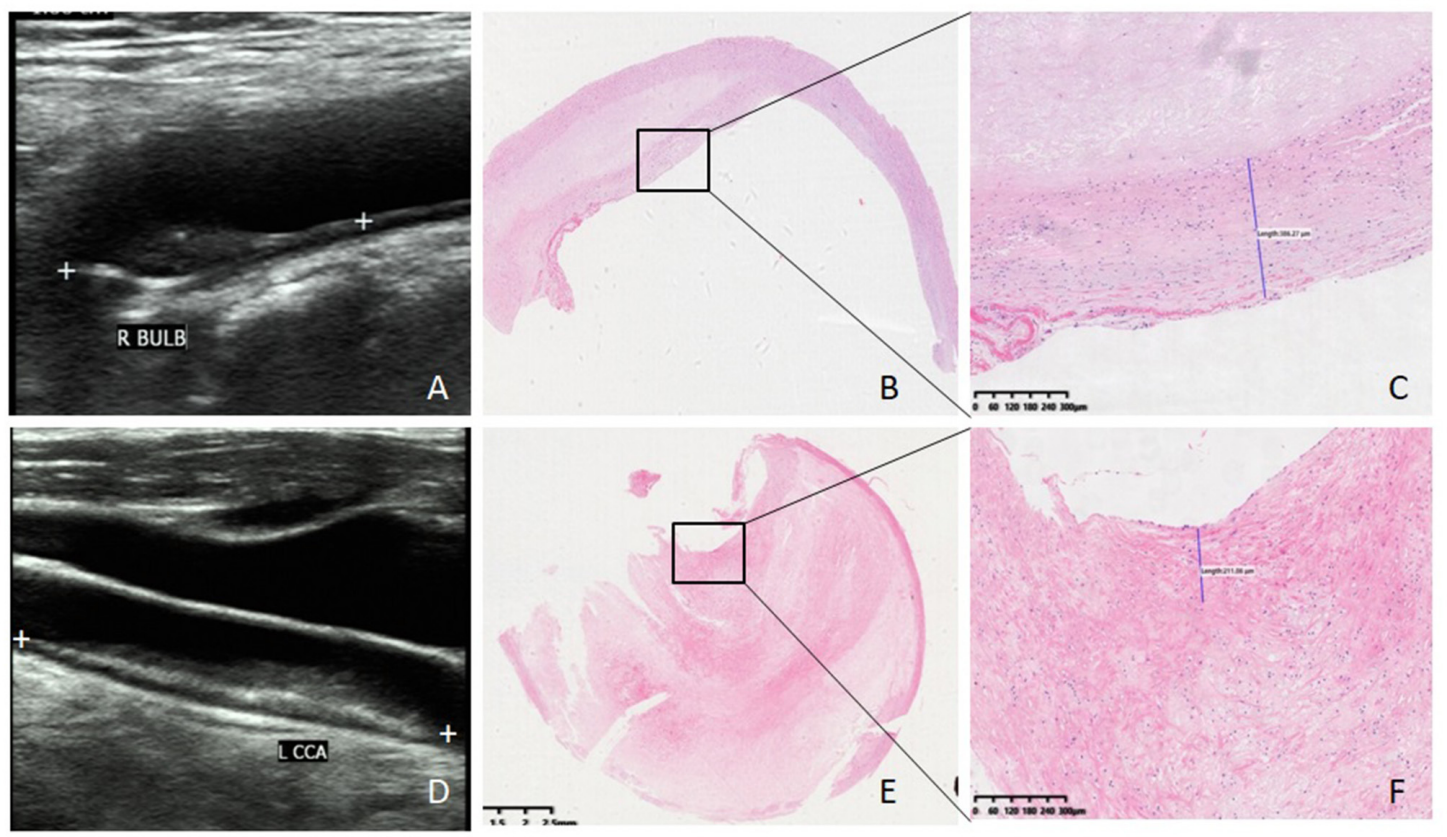
Ultrasound imaging, HE staining, and fibrous cap thickness of plaque. **(A)** Ultrasound image of stable plaque (white plus signs demonstrate a hypoechoic plaque with regular shape and scored two). **(B)** HE staining of stable plaque (×20). **(C)** Fibrous cap thickness of the same stable plaque 386 μm (×100). **(D)** Ultrasound image of vulnerable plaque (white plus signs demonstrate a mix-echoic plaque with stenosis rate 80% and scored four). **(E)** HE staining of vulnerable plaque ( ×20). **(F)** fibrous cap thickness of the same vulnerable plaque 211 μm (×100).

A standard carotid artery ultrasound examination was conducted using a linear array probe L9-3 of Philips IU-Elite scanner (Philips Medical System, Washington). The patient was positioned in a supine position with the head turning to the side contralateral to the carotid examined. The mentioned features of plaque were observed or measured: shape, echogenicity, the existence of ulcer or not (including incomplete of plaque's fibrous cap), vascular luminal stenosis rate, size (length and thickness), and resistance index (RI) in the narrow place. Past studies proved that the following ultrasound features could tend to vulnerable plaque: irregular shape, hypoechoic, ulcer, vascular luminal stenosis rate (9, 10, 13, 14). Therefore, each carotid artery plaque was scored as follows: shape (regular 0, irregular 1); major echogenicity (hyperechoic 0, mixed 1, and hypoechoic 2); ulcer (none 0, existence 1); vascular luminal stenosis rate (<50% 0, 50–69% 1, 70–89% 2, >90% 3) Figure 1. Then each plaque was separated into upstream segment and downstream segment, and the score was assessed for each part. The standard carotid artery ultrasound examinations were operated by the same technician with 6 years of experience and all images were independently assessed by two sonologists blinded to the information of the patients.

### Pathological Analysis

All the specimens contained the entire plaque, the entire intima, and a part of the media of the carotid artery wall. They were fixed in formalin immediately and decalcified in EDTA buffer. The upstream shoulder and downstream shoulder of each plaque were cut transversely at 5 mm thickness and embedded in paraffin. Serial transverse sections of 4 μm thickness were stained for hematoxylin-eosin staining and Sirius red staining.

All plaques were classified into two groups (vulnerable group and stable group) by histological features according to the following criteria: the plaque with at least one major criterion or two minor criteria was classified to the vulnerable group, or otherwise to the stable group (9). Major criteria were as follows: 1. active inflammation, 2. thin cap with a large lipid core, 3. endothelial denudation with superficial platelet aggregation, 4. fissured plaque, and 5. stenosis >90%. Minor criteria were as follows: 1. superficial calcified nodule, 2. glistening yellow, 3. intraplaque hemorrhage, 4. endothelial dysfunction, and 5. positive remodeling Figure 1.

In addition, the fibrous cap thickness of carotid artery plaque was also measured and recorded Figure 1.

All the plaques were analyzed, and fibrous caps were measured by two histopathologists independently who were blinded to the information and ultrasound results of the patients.

### Sirius Red–Picric Acid Staining and Collagen Features of Plaque

The transverse sections of each plaque (4 μm thickness) were stained for Sirius red–picric acid (Bry 0013, Ruiyu Tech, shanghai, China). The sections were routinely dewaxed and washed using distilled water. Then, they were soaked in the Sirius red–picric acid dye for 30 min and washed using dehydrated alcohol three times.

**Figure 2:**
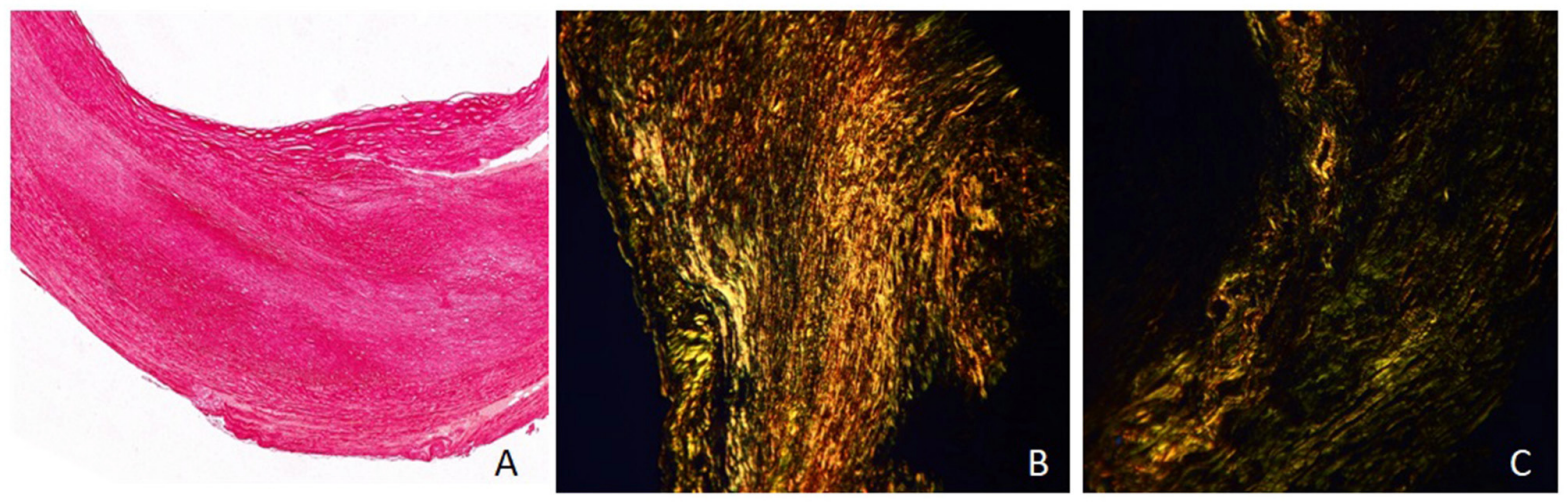
Sirius red–picric acid staining and collagen features of plaque (×40). **(A)** Collagen tissue showed red under the conventional optical microscope. **(B)** Under the polarized light microscope, collagen type I showed yellow or red intense birefringence (area ratio: 78%). **(C)** Under the polarized light microscope, collagen type III showed thin fibril with weak greenish birefringence (area ratio: 43%), and type VI showed thin fibril with weak yellow birefringence (area ratio: 35%).

The collagen tissue was stained into red under the conventional optical microscope. Under the polarized light microscope, the different types of collagens were shown in different colors because of the birefringence characteristics: type I showed yellow or red intense birefringence, type III showed thin fibril with weak greenish birefringence, and type VI showed thin fibril with weak yellow birefringence (Figure 2).

All the sections were observed both under the conventional optical microscope and under the polarized light microscope at 40 times the vision field. Three vision fields under the polarized light microscope were randomly chosen and recorded by photographs in each section. All images were analyzed by Image-pro plus (version 6.0). The collagen features were assessed as follows: the area ratio of collagen to plaque area and the area ratio of different types of collagen (I, III, IV) to all collagen. The mean values of three images of each section were recorded as the parameters of each plaque.

### Statistical Analysis

Statistical analysis was conducted by SPSS software (version 19; SPSS Inc., United States). Measured data (length, thickness, vascular stenosis rate, resistance index, thickness of fibrous cap, ratio of collagen to plaque area, and ratio of different types of collagen to plaque area) were presented as the mean value ± SD, and *P*-value < 0.05 was considered statistically significant.

All the measured data of between-group comparisons were performed by the Shapiro–Wilk test (W test) to assess for normal distributions. Normally distributed measured data were analyzed using one-way ANOVA. Non-normally distributed data were analyzed using the Mann–Whitney U test. The enumeration data were performed by chi-squared test. The comparison of the ratio of collagen to plaque area in upstream shoulders and downstream shoulders was analyzed by paired-samples *t*-test, as well as the ratio of different types of collagen to plaque area. The comparison of ultrasound scores between the vulnerable group and the stable group was analyzed by the Mann–Whitney U test. The comparison of ultrasound scores between the upstream shoulder and downstream shoulder was analyzed by Wilcoxon signed-rank test. Receiver-operating characteristic curves (ROCs) for ultrasound scores were performed to evaluate diagnostic performance. Further, optimal cutoff value, the corresponding sensitivity, specificity, and accuracy of ultrasound score were calculated. The correlation analysis was conducted by Pearson's correlation or Spearman's rank correlation. The interobserver agreements of ultrasound score and pathological classification were assessed by Cohen's kappa statistics.

## Results

Fifty-five carotid artery plaques met the inclusion criteria. Among them, four carotid plaques were excluded by the exclusion criteria above (one was the lack of complete serum test information, one was fragmented, and two were failed to be dyed by Sirius red–picric acid staining). Finally, a total of 51 plaques were included in the final analysis.

### Ultrasound Features, Fibrous Cap, and Collagen Features of the Vulnerable Group and the Stable Group

Fifty-one plaques were classified into two groups according to the pathological features: 40 in the vulnerable group and 11 in the stable group. The clinical features are displayed in Table 1. The symptoms of stoke in 6 months before CEA were more likely to happen in the vulnerable group than in the stable group (*P* = 0.026).

**Table 1:** Clinical features in patients (*n* = 51).

**Subject characteristics**	**Vulnerable group**	**Stable group**	***P*-value**
Number	40	11	/
Age (year)	61.4 ± 8.1	66.1 ± 7.8	0.265
Male/Female (*n*)	38/2	10/1	0.526
BMI (kg/m^2^)	23.5 ± 5.8	21.7 ± 5.5	0.146
Smoking (≥1/day, ≥1year) (*n*) (%)	37 (92.5)	9 (81.8)	0.292
Hypertension (≥140/90 mmHg) (*n*) (%)	35 (87.5)	8 (72.7)	0.468
Diabetes (*n*) (%)	26 (65)	7 (63.6)	0.933
Symptoms of stroke in 6 months (*n*) (%)	28 (70)	3 (27.2)	0.026^*^
Statin treatment (*n*) (%)	35 (67.3)	7 (63.6)	0.164
Family history of stroke (*n*) (%)	10 (19.2)	2 (18.2)	0.944
Total cholesterol (mmol/L)	3.8 ± 0.7	3.7 ± 0.7	0.751
Triglycerides (mmol/L)	1.4 ± 0.5	1.3 ± 0.5	0.628
HDL-C (mmol/L)	1.0 ± 0.5	1.1 ± 0.4	0.582
LDL-C (mmol/L)	2.2 ± 0.8	1.9 ± 0.7	0.193
Hs-CRP (mg/L)	4.7 ± 4.6	3.8 ± 4.7	0.082
Fibrinogen (g/L)	3.2 ± 1.3	3.8 ± 1.2	0.072
Lithic acid (μmol/L)	315.4 ± 36.1	285.4 ± 31.2	0.103

*HDL-C, High-density lipoprotein cholesterol; LDL-C, Low-density lipoprotein cholesterol; Hs-CRP, High-sensitive C-reactive protein*.

* *Significant difference (P < 0.05)*.

Table 2 shows the ultrasound features of the two groups. The ultrasound scores of the vulnerable group were significantly higher than those of the stable group (4.35 ± 1.23 vs. 2.09 ± 1.04, *P* = 0.001). ROC curve analysis showed that the AUC for ultrasound score was 0.894 (95% CI: 0.795, 0.994; *P* < 0.001) in differentiating vulnerable plaque and stable plaque. The best cutoff point was three. The sensitivity, specificity, positive predictive value, and negative predictive value were 87.5, 72.7, 92.1, and 61.5% separately. Youden's index was 0.602.

**Table 2:** Ultrasound features and pathological features of two groups.

**Features**	**Vulnerable group**	**Stable group**	***P*-value**
Number of plaques	40	11	/
**Ultrasound features**			
Ultrasound scores	4.35 ± 1.23	2.09 ± 1.04	0.001^*^
Length of plaques (mm)	43.7 ± 14.3	25.7 ± 8.6	0.064
Thickness of plaques (mm)	4.8 ± 1.3	3.1 ± 0.6	0.001^*^
Vascular luminal stenosis rate (%)	75.5 ± 19.1	62.5 ± 12.8	0.046^*^
Resistance index (in the narrow place of vessel)	0.83 ± 0.18	0.78 ± 0.15	0.462
**Pathological features**			
Fibrous cap thickness (μm)	185.6 ± 64.3	322.8 ± 95.7	0.012^*^
Area ratio of all collagen to plaque (%)	45.6 ± 17.4	67.4 ± 25.8	0.005^*^
Area ratio of collagen type I to all collagen (%)	55.2 ± 13.8	69.5 ± 17.5	0.033^*^
Area ratio of collagen type III to all collagen (%)	23.5 ± 9.6	20.3 ± 8.1	0.381
Area ratio of collagen type IV to all collagen (%)	21.7 ± 8.5	13.4 ± 6.4	0.026^*^

* *Significant difference (P < 0.05)*.

The plaques of the vulnerable group were significantly thicker than those of the stable group (4.8 ± 1.3 vs. 3.1 ± 0.6, *P* = 0.001). The vascular luminal stenosis rates of the vulnerable group were significantly higher than those of the stable group (75.5 ± 19.1 vs. 62.5 ± 12.8, *P* = 0.046). Otherwise, the length of plaque and RI in the narrow place of the vessel were not significantly different in the two groups.

**Figure 3:**
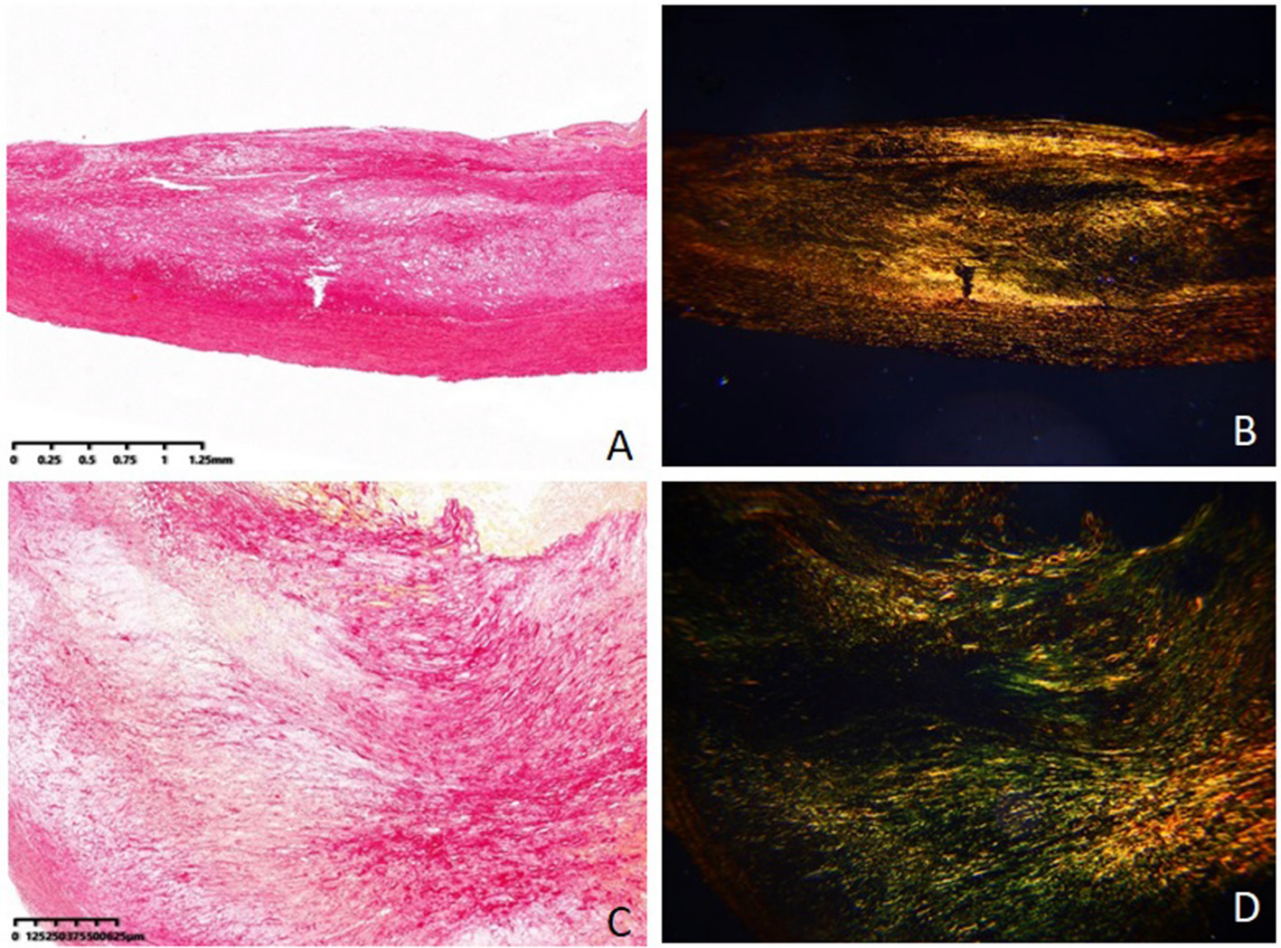
Collagen tissue in stable plaque and vulnerable plaque (Sirius red–picric acid staining, ×40). **(A)** Collagen tissue in stable plaque under the conventional optical microscope. **(B)** Collagen tissue in stable plaque under the polarized light microscope. **(C)** Collagen tissue in vulnerable plaque under the conventional optical microscope. **(D)** Collagen tissue in vulnerable plaque under the polarized light microscope.

Table 2 also shows the pathological features of the two groups. The fibrous caps of the vulnerable group were significantly thinner than those of the stable group (185.6 ± 64.3 vs. 322.8 ± 95.7, *P* = 0.012) (Figure 2). The area ratios of all collagen to plaque in the vulnerable group were significantly lower than in the stable group (45.6 ± 17.4 vs. 67.4 ± 25.8, *P* = 0.005). The area ratios of collagen type I to all collagen in the vulnerable group were significantly lower than in the stable group (55.2 ± 13.8 vs. 69.5 ± 17.5, *P* = 0.033). The area ratios of collagen type IV to all collagen in the vulnerable group were significantly higher than in the stable group (21.7 ± 8.5 vs. 13.4 ± 6.4, *P* = 0.026). Otherwise, the area ratios of collagen type III to all collagen were not significantly different in the two groups (*P* = 0.381) (Figure 3).

### Ultrasound Features, Fibrous Cap, and Collagen Features of Plaque's Upstream Shoulder and Downstream Shoulder

The ultrasound scores and pathological features of the upstream shoulder and downstream shoulder are shown in Table 3.

**Table 3:** Ultrasound score and pathological features of upstream shoulder and downstream shoulder.

**Features**	**Upstream shoulder**	**Downstream shoulder**	***P*-value**
Ultrasound scores	3.55 ± 1.48	2.96 ± 1.33	0.001^*^
Fibrous cap thickness (μm)	142 ± 73.9	368 ± 103.9	0.001^*^
Area ratio of all collagen to plaque (%)	52.6 ± 19.8	63.4 ± 29.3	0.032^*^
Area ratio of collagen type I to all collagen (%)	54.9 ± 15.9	68.5 ± 19.1	0.022^*^
Area ratio of collagen type III to all collagen (%)	23.2 ± 8.9	21.3 ± 9.1	0.583
Area ratio of collagen type IV to all collagen (%)	26.7 ± 9.5	15.4 ± 7.4	0.016^*^

* *Significant difference (P <0.05)*.

The ultrasound scores of plaque's upstream shoulders were significantly higher than those of downstream shoulders (3.55 ± 1.48 vs. 2.96 ± 1.33, *P* = 0.001). The fibrous caps of plaque's upstream shoulders were significantly thinner than those of downstream shoulders (142 ± 73.9 vs. 368 ± 103.9, *P* = 0.001). The area ratios of all collagen to the plaque of upstream shoulders were significantly lower than those of downstream shoulders (52.6 ± 19.8 vs. 63.4 ± 29.3, *P* = 0.032). The area ratios of collagen type I to all collagen of upstream shoulders were significantly lower than those of downstream shoulders (54.9 ± 15.9 vs. 68.5 ± 19.1, *P* = 0.022). The area ratios of collagen type IV to all collagen of upstream shoulders were significantly higher than those of downstream shoulders (26.7 ± 9.5 vs. 15.4 ± 7.4, *P* = 0.016). Otherwise, the area ratios of collagen type III to all collagen were not significantly different in the two groups (*P* = 0.583).

### Correlation Analysis of Ultrasound Score, Fibrous Cap Thickness, and Collagen Area Ratio

We found a significant direct relationship between ultrasound score and area ratios of collagen type IV to all collagen (*P* = 0.036, correlation coefficient: 0.83). On the other hand, the ultrasound scores of the vulnerable group were significantly higher than those of the stable group. Both of the results suggest that collagen type IV can present the severity of plaque's vulnerability partly.

We also found a significant direct relationship between fibrous cap thickness and area ratios of collagen type I to all collagen (*P* = 0.046, correlation coefficient: 0.67).

### Interobserver Agreement

The Cohen's kappa value between two observers for ultrasound score was 0.86 (95% CI = 0.77–0.95), 0.92 (95% CI = 0.85–0.98) for pathological classification and 0.83 (95% CI = 0.74–0.92) for fibrous cap thickness measurement.

## Discussion

Because vulnerable carotid artery plaques are one of the most important risk factors for acute stroke, we aimed to assess whether carotid artery ultrasound score could have a clinical value in identifying vulnerable plaque and the impacts of collagen distribution on the stability of plaques.

We scored all the plaques by ultrasound features (0–7) and classified them into the vulnerable group and the stable group by pathological features. The results showed that the ultrasound scores of the vulnerable group were significantly higher than those of the stable group (4.35 ± 1.23 vs. 2.09 ± 1.04, *P* = 0.001), and the AUC of ultrasound score was higher than 0.7 in differentiating vulnerable plaques from stable plaques (AUC = 0.894, best cutoff point three), which prompted that ultrasound score had a clinical application value in identifying vulnerable plaques.

We also analyzed collagen distribution features in plaque, because collagen is the major extracellular component of artery plaque, comprising 30% of the dry weight and up to 60% of the total protein content of advanced human vascular lesions (15, 16). Our study showed that the area ratios of all collagen to plaque in the vulnerable group were significantly lower than in the stable group (*P* = 0.005). This result proved that the reduction of collagen in the plaque was one of the characteristics of vulnerable plaque (9). The major collagen type of advanced lesions is type I, which represents about 70% of the total collagen. We found the area ratios of collagen type I to all collagen in the vulnerable group were significantly lower than in the stable group (55.2 ± 13.8 vs. 69.5 ± 17.5, *P* = 0.033). The ratios of stable plaques were similar to the previous researches (17, 18). Otherwise, the area ratios of collagen type IV to all collagen in the vulnerable group were significantly higher than in the stable group (*P* = 0.026) and a significant direct relationship between ultrasound score and area ratios of collagen type IV to all collagen (*P* = 0.036, correlation coefficient: 0.83) could be found, which prompted that type IV collagen was also increased in vulnerable plaques (19).

The fibrous cap plays an important role in plaque stability. The thickness of the cap is one of the major criteria of vulnerable pathological criteria (20). The results showed that the fibrous caps of the vulnerable group were significantly thinner than those of the stable group (*P* = 0.012), which proved that the thin cap could affect the stability of plaque. Besides, collagen is the major component of the fibrous cap. The fibrous cap thickness results were also in accord with the collagen results. A significant direct relationship between fibrous cap thickness and area ratios of collagen type I to all collagen was found. All these results revealed that collagen type I played an important role in the stability of plaque: the more collagen type I, the thicker the fibrous cap and the more stable the plaque could be. In addition, collagen type I under microscope represents the birefringence more intense than type III and type IV. This result perhaps explained why the higher the ratios of collagen type I, the more stable the fibrous cap and the plaque could be.

Further analysis showed that ultrasound scores of plaque's upstream shoulders were significantly higher than those of downstream shoulders, which prompted that the upstream shoulder could be more vulnerable than the downstream shoulder. These results were in keeping with the results of the fibrous cap (the fibrous caps of plaque's upstream shoulders were significantly thinner than those of downstream shoulders). Otherwise, the results of collagen ratios also proved that the upstream shoulder could be more vulnerable (compared with the downstream shoulder, the area ratios of all collagen to the plaque of upstream shoulders were significantly lower; the area ratios of collagen type I to all collagen of upstream shoulders were significantly lower; the area ratios of collagen type IV to all collagen of upstream shoulders were significantly higher). These results could explain why the upstream shoulders of plaque had more ruptures than the downstream shoulders and they also reminded the radiologists to pay more attention to the upstream shoulder in preoperational examinations.

This study had some pities and limitations. First, most patients in this study had medicine treatment before ultrasound examination and CEA operation, so the results of the analysis on serum examinations were not accurate. Second, the ultrasound features and collagen features of other parts of plaques, like peak, should be analyzed later. Third, the collagen ratios measured in this study were area ratio instead of weight ratio, which could have limitations in results analysis. Fourth, further studies on the other components in the plaque that might act on plaque stability could be conducted.

In conclusion, a carotid artery ultrasound score could have a clinical value in identifying vulnerable plaque and the collagen distribution could impact the stability of plaques, especially collagen type I and type IV. In addition, both the ultrasound score and collagen distribution prompted that the upstream shoulders were more vulnerable than the downstream shoulders, which reminded that more attention should be paid to the upstream shoulder during the preoperational ultrasound examination.

## Data Availability Statement

The original contributions presented in the study are included in the article/supplementary material, further inquiries can be directed to the corresponding author.

## Ethics Statement

The studies involving human participants were reviewed and approved by First Affiliated Hospital of Soochow University. The patients/participants provided their written informed consent to participate in this study. Written informed consent was obtained from the individual(s) for the publication of any potentially identifiable images or data included in this article.

## Author Contributions

RH: study design data analysis and writing. YY and YD: data collection and analysis. YH and PZ: data collection. PH: study design and writing. All authors contributed to the article and approved the submitted version.

## Funding

Our research was funded by Science and Technology of People's Livelihood in Suzhou City-Research on the Application of Key Technologies (No. SS202061), Shanghai Jiao Tong University Star of Jiao Tong University Program Medical and Industrial Cross Research Fund (No. YG2021QN31), Cadre Health Care Research Project of Jiangsu Province (No. BJ17010) and People's Livelihood Science and Technology Demonstration Project of Suzhou (No. SS201859).

## Conflict of Interest

The authors declare that the research was conducted in the absence of any commercial or financial relationships that could be construed as a potential conflict of interest.

## Publisher's Note

All claims expressed in this article are solely those of the authors and do not necessarily represent those of their affiliated organizations, or those of the publisher, the editors and the reviewers. Any product that may be evaluated in this article, or claim that may be made by its manufacturer, is not guaranteed or endorsed by the publisher.
