# Corrigendum: The correlation between collagen types and ultrasound feature score in evaluating the vulnerability of carotid artery plaque

**DOI:** 10.3389/fcvm.2023.1187049

**Published:** 2023-03-31

**Authors:** Ruijun Han, Yanhong Yan, Yafang Ding, Yabo Huang, Peng Zhou, Pinjing Hui

**Affiliations:** ^1^ Department of Stroke Center, The First Affiliated Hospital of Soochow University, Suzhou, China; ^2^ Department of Ultrasound, Ren Ji Hospital, Shanghai Jiao Tong University School of Medicine, Shanghai, China

**Keywords:** collagen, arteriosclerosis, plaque, ultrasound, carotid artery

A Corrigendum on The correlation between collagen types and ultrasound feature score in evaluating the vulnerability of carotid artery plaque By Han R, Yan Y, Ding Y, Huang Y, Zhou P and Hui P. (2021) Front. Cardiovasc. Med. 8:756424. doi: 10.3389/fcvm.2021.756424

In the published article, there was an error in the Funding statement. The order of funding statements should be changed. The correct Funding statement appears below.

**FUNDING**

Our research was funded by science and technology of people's livelihood in Suzhou city-research on the application of key technologies (No. SS202061), Shanghai Jiao Tong university star of Jiao Tong university program medical and industrial cross research fund (No. YG2021QN31), cadre health care research project of Jiangsu Province (No. BJ17010) and people's livelihood science and technology demonstration project of Suzhou (No.SS201859).

The authors apologize for this error and state that this does not change the scientific conclusions of the article in any way. The original article has been updated.

